# The feasibility of using life skills training in primary schools to improve mental health and academic performance: a pilot study in Kenya

**DOI:** 10.1186/s12888-022-03781-x

**Published:** 2022-02-17

**Authors:** David M. Ndetei, Victoria N. Mutiso, Christine W. Musyimi, Rita K. Alietsi, Jenelle R. Shanley, Kamaldeep S. Bhui

**Affiliations:** 1 Africa Mental Health Research and Training Foundation, Nairobi, Kenya; 2 Department of Psychiatry, University of Nairobi, Nairobi, Kenya; 3 World Psychiatric Association Collaborating Centre for Research and Training, Nairobi, Kenya; 4 School of Graduate Psychology, Pacific University, Forest Grove, USA; 5 Department of Psychiatry, University of Oxford, Oxford, England; 6 World Psychiatric Association Collaborating Centre for Research and Training, London, UK

**Keywords:** Life skills training, Primary school children, Academic performance, Kenya

## Abstract

**Background:**

There is no Kenyan evidence on the relationship between mental illness and academic performance. We aimed to determine the effect of life skills training on mental health and academic performance.

**Methods:**

We administered to 1848 primary school children a researcher designed socio-demographic questionnaire, and the Youth Self Report (YSR) and Child Behavior Checklist (CBCL) to their parents, followed by eight sessions of life skills training. We extracted data from the individual records of each child on overall performance pre and post training separated by one year. We conducted descriptive statistics, paired sample t-tests, multivariate linear regression analysis and linear mixed model analysis to assess changing patterns of academic performance and any predictive characteristics.

**Results:**

There was significant (p < 0.05) improvement in overall academic performance (aggregate marks and all individual subjects) for both lower primary and upper primary classes after the life-skills training intervention. For lower classes (2-4 grades) increase in academic performance was significantly associated with fathers and mothers education levels, region and class. For upper classes, (5-7 grades) increase in academic performance was associated with region, class and age.

**Conclusions:**

Life skills training is recommended as it could improve academic performance, but predicted by socio-demographic factors.

## Highlights

- School children.
- Mental health.
- Life skills training.
- Academic performance.

## Introduction

Evidence, mainly in high income countries (HICs) demonstrates a relationship between children’s mental disorders and poor academic performance: poor concentration [1, 2]; depression, conduct disorders, substance use [1]; and anxiety or school phobia [3] and suicidality [4]. The greater the number of conditions, the poorer the academic performance [5, 6]. Mental disorders are also associated with increased school drop-out [7–9], and truancy [10] due to psychosocial dysfunction [11] and perception of poor academic performance leading to low self-esteem. [12]. A Dutch study found that the negative effect of externalizing problems on academic achievement was not attributable to the Intelligent Quotient (IQ) of the children i.e. not to their intellectual potential but to other factors [13]. There is a dearth of literature on comparable studies in LMIC, creating a gap in our understanding on how HIC and LMIC compare and contrast. This study seeks to contribute towards addressing this gap. The finding from the Dutch study that factors other than 1Q alone contribute to externalizing problems calls for context appropriate determination of other possible contributors to externalizing behavior, not only in a HIC but also in LMIC.

School based approach to mental health can improve academic performance [14, 15]. It can also increase the chance of the students remaining in school [16]. Mental health disorders are highly prevalent ranging from 12 to 37% depending on the type of condition in school going children in Africa, for example South Africa [17] and Kenya [18–21]. These prevalence rates are similar to that found in HICs such as the USA [22]. These comparable rates in prevalence of mental disorders in youth in both HIC and LMIC is further justification to study how mental disorders are related to academic performance in LMIC as compared to HIC. A further justification is the importance of intervention as early as possible. Preliminary data shows that life skills training reduces the level of symptoms on Youth Self-Report (YSR) scores in primary school children in Kenya [23]. This study aims to test the feasibility of applying life skills training for schools in a Kenyan setting with the view to treat mental disorders and enhance academic performance. This information can inform policy and practice on school mental health in Kenya and other similar settings.

## Methods

### Study design

This was a one group pre-post intervention study design implemented as a program.

### Study Site

This study was part of a bigger study titled The Kenya Integrated Intervention Model for Dialogue and Screening to Promote Children’s Mental Wellbeing (KIDS). The study had identified Makueni and Machakos counties in South East Kenya. Makueni is predominantly rural whereas Machakos has large peri-urban population. In Makueni County, we randomly picked Makindu sub-county, one of the 6 sub-counties in Makueni County and in Machakos County we randomly picked Machakos sub-county. For the purpose of this study, Machakos sub-county is referred to as peri-urban study site whereas Makindu sub-county is referred to as rural study site. These two counties were chosen for KIDS because Africa Mental Health Research and Training Foundation (AMHRTF) had been undertaking community mental health implementation research at the invitation of the local County Governments. School mental health had not been part of this community mental health. In order to facilitate effective supervision of schools by the school supervisors, the schools in each of sub-county are divided into several groups; each group being referred to by MoE as a cluster. We randomly chose six clusters per sub-county and then randomly selected two schools per cluster to meet our predetermined sample. We sampled a total of 23 schools, 11 from Machakos sub-county and 12 from Makindu sub-county.

### Study Participants

Participants were primary school children and their parents in the participating lower primary (classes 2-4 aged 7 - 10 years) and upper primary (classes 5-7 aged 11 - 13 years) schools. The required school entry age in Kenya is 6 years. Because we wanted to have both the end of year examination results for the previous year and the current end of year, we excluded performance analysis on first year (class 1) who did not have any results and final year (class 8) because of preparation for the National Examinations which are different from those end of year examinations that are administered by the schools.

### Measures

#### The Socio-Demographic Questionnaire

We used a researcher-designed socio-demographic questionnaire, completed by the children on themselves and also on their parents to include age, gender, region (rural or peri-urban) and class for the pupils, and parents marital status, employment status and education level.

##### Mental Health Assessment

The Youth Self-Report (YSR) has good psychometric properties [24] and has been used across different and multicultural societies [25] by children aged 11-18 years. It is a self-report on mental disorders. The Child Behavior Checklist (CBCL) is administered to parents or caretakers to report mental health problems among children 6 to 18 years [26]. Both YSR and CBCL focus on the previous 2 weeks at the time of administration. [24, 26] have broken down the syndromes of CBCL and YSR, the summary scores and how to interpret the scores as follows; (i) The syndromes - Anxious/Depressed, Withdrawn/Depressed, Somatic Complaints, Social Problems, Thought Problems, Attention Problems, Rule-Breaking Behavior and Aggressive Behavior. (ii) The summary scores - Internalizing Problems (summarizing the Anxious/Depressed, Withdrawn/Depressed, and Somatic Complaints scores), Externalizing Problems (summarizing the Rule-Breaking Behavior and Aggressive Behavior scores) and Total Problems score (summarizing all 8 syndrome scale scores) and (iii) the interpretation of the scores - Each scale score is interpreted based on the *T* score whereby *T* score of (below 65) is considered to be in the normal range, (65 to 69) is considered to be in the borderline range and (70 and above) is considered to be in the clinical range.

##### Academic Performance

The Kenya Institute of Curriculum Development provides guidelines on subjects to be examined and how to grade them [27]. The subjects examined in the primary schools were as follows: Mathematics, English, Kiswahili, Science and Social Studies where each subject is worth a maximum of 100 marks and yielding a maximum aggregate of 500 marks for all subjects. The grading system was based on an expanded 12- letter grade ranging from A (highest) to F (Lowest). The subject letter grades and their corresponding scores were A (80-100), A-(75-79.99), B+(70-74.99), B(65-69.99), B-(60-64.99), C+(55-59.99), C(50-54.99), C-(45-49.99), D+(40-44.99), D(35-39.99), D-(30-34.99) and F(0-29.99). Alternatively, the aggregate letter grades and their corresponding marks were A(400-500), A-(375-399), B+(350-374), B(325-349), B-(300-324), C+(275-299), C(250-274), C-(225-249), D+(200-224), D(175-199), D-(150-174) and F(0-149). Cumulative end of year scores were collected for each student for the individual subject and an aggregate for all subjects. The baseline was the end of year scores before the intervention and the post-intervention score was on end of year scores in the following year. We extracted these scores from the school transcripts that normally provide this information. This extraction was part of the data collection and done at around the same time we were administering the other instruments.

##### The Ministry of Education (MoE) Life Skills Training Curriculum

The intervention took place early in the second term (May and June). The intervention focused on life skills training, using the standard life-skills training curriculum developed by MoE [28] with the help of expert consultants provided by World Health Organization (WHO) and United Nations International Children’s Emergency Fund (UNICEF) to the MoE Department of Curriculum Development. The joint MoE/UNICEF expert committee adapted the WHO life skills program [29] to the Kenyan socio-cultural context. The adapted curriculum had two versions; one for lower primary school (standard1-4) and one for upper primary school (standard 5-8). It was then piloted and adopted as the official standard life skills curriculum for all schools. It was designed to fit into the eight hours that are allowed for extra-curriculum activities, as part of all other curriculums by the MoE. The training was spread out with flexibility to suit the convenience of individual schools. We negotiated with each school and class the best time to train their children for the eight hours spread equally over four weeks. Thus, this curriculum has an inbuilt time line (a total of 8 h spread out to suit the convenience of each school) and has well-structured curriculum content covered in a systematic manner. Thematic lines of the adapted curriculum included: critical/creative thinking, effective communication, empathy, decision making, stress management and internal locus of control. Each student has their own booklet on items to be covered and a check out for completed skills. This approach ensured that every student in this study completed the 8 h, using a confirmed content for all students and same level of quality of fidelity of the training. This project facilitated every child in the study sites to have their own copy of the booklet so as to follow using their own copy what they were being trained by our consultant specifically hired for this project.

##### Study Procedures

We used consultant trainers. These trainers were professional teachers who had taken further training on life-skills and are used to train teachers, but we also used them to conduct life-skills training for the children. We sought consent from the parents during one of the parent-teacher meetings. We explained the nature of the study, the potential benefits of improving the mental health of the children and the academic performance in the children that there were no risks involved and that it was voluntary. We explained that we will conduct the interventions early in the second term (May and June) and that we will avoid the third term, which starts in September, because of the schools’ preparations for end of year examinations. All the parents consented and signed for their respective children. This was repeated for the children in the classroom situation.

### Statistical analysis

We used descriptive statistics to determine socio-demographic characteristics (as summarized in Table 1) of the sample as well as CBCL and YSR syndrome scales; paired sample t-tests to assess the differences before and after intervention on the aggregate academic performance scores as well as the individual subject’s scores; linear mixed model to determine predictors of academic performance controlling for socio demographic characteristics on improvement and mental disorders. All the analyses were done using STATA version 14.

## Results

Table 1 summarizes the socio-demographics of the children and their parents divided by lower and upper primary children, including the CBCL/YSR syndrome mean scores at pre- and post-intervention and the follow up completion rates (85-95%) for each variable. Only a small number of the participants were lost to follow-up while the majority were in the study from pretest to posttest as seen from the follow-up rates. The mean scores for attention, internalizing, externalizing and total scores were comparable for both lower and upper classes in pre and posttest. Fathers education level, Mothers education level, region and class are significantly associated with increase in academic performance in lower class while region, class and age are significantly associated with increase in academic performance in upper class.

**Table 1:** Demographic Characteristics of Participants and follow-up Rates

Variable	Category	Lower Class (2-4)					Upper Class (5-7)				
		Pretest	Posttest	Follow-up Rate (%)	F	P-Value^††^	Pretest	Posttest	Follow-up Rate (%)	F	P-Value^††^
		*N* = 1072	*N* = 976				*N* = 776	*N* = 697			
		n(%)	n(%)	91.0			n(%)	n(%)	89.8		
Gender	Female	534(49.8)	491(50.3)	91.9	0.3045	0.9345	394(50.8)	354(50.8)	89.8	1.3355	0.2396
	Male	538(50.2)	485(49.7)	90.1			382(49.2)	343(49.2)	89.8		
Guardianship	Both parents	800(74.6)	727(74.5)	90.9	0.6974	0.7551	596(76.8)	544(78.0)	91.3	1.2114	0.2698
	Single parent	158(14.7)	143(14.7)	90.5			113(14.6)	94(13.5)	83.2		
	Others	66(6.2)	61(6.3)	92.4			65(8.4)	57(8.2)	87.7		
	Missing	48(4.5)	45(4.6)				2(0.3)	2(0.3)			
Marital status of the parents	Married	872(81.3)	793(81.3)	90.9	1.8915	0.0806	618(79.6)	564(80.9)	91.3	0.9921	0.4299
	Single/divorced	121(11.3)	108(11.1)	89.3			137(17.7)	113(16.2)			
	Missing	79(7.4)	75(7.7)				21(2.7)	20(2.9)	95.2		
Fathers education level	Primary or less	156(14.6)	135(13.8)	86.5	2.3479	0.0057	240(30.9)	210(30.1)	87.5	1.1111	0.3470
	Secondary or more	384(35.8)	345(35.3)	89.8			335(43.2)	303(43.5)	90.4		
	Don’t know	411(38.3)	383(39.2)	93.2			169(21.8)	155(22.2)	91.7		
	Missing	121(11.3)	113(11.6)				32(4.1)	29(4.2)			
Mothers education level	Primary or less	199(18.6)	177(18.1)	88.9	2.5989	0.0021	334(43.0)	302(43.3)	90.4	1.2707	0.2303
	Secondary or more	342(31.9)	305(31.3)	89.2			299(38.5)	265(38.0)	88.6		
	Don’t know	445(41.5)	414(42.4)	93.0			132(17.0)	119(17.1)	90.2		
	Missing	86(8.0)	80(8.2)				11(1.4)	11(1.6)			
Fathers employment	Unemployed	263(24.5)	240(24.6)	91.3	1.7293	0.1123	230(29.6)	211(30.3)	91.7	0.2899	0.9417
	Employed	672(62.7)	611(62.6)	90.9			489(63.0)	435(62.4)	89.0		
	Missing	137(12.8)	125(12.8)	91.2			57(7.3)	51(7.3)	89.5		
Mothers employment	Unemployed	704(65.7)	643(65.9)	91.3	0.3721	0.8967	422(54.4)	381(54.7)	90.3	1.4625	0.1893
	Employed	284(26.5)	255(26.1)	89.8			327(42.1)	293(42.0)	89.6		
	Missing	84(7.8)	78(8.0)				27(3.5)	23(3.3)			
Region	Peri-urban	593(55.3)	547(56.0)	92.2	5.9956	<0.0001	391(50.4)	351(50.4)	89.8	9.8356	<0.0001
	Rural	479(44.7)	429(44.0)	89.6			385(49.6)	346(49.6)	89.9		
Class	Class 2 (5)	263(24.5)	251(25.7)	95.4	4.2580	<0.0001	309(39.8)	270(38.7)	87.4	3.8912	<0.0001
	Class 3 (6)	254(23.7)	225(23.1)	88.6			376(48.5)	346(49.6)	92.0		
	Class 4 (7)	286(26.7)	244(25.0)	85.3			88(11.3)	78(11.2)	88.6		
	Missing	51(4.8)	48(4.9)				3(0.4)	3(0.4)			
Age in years	Mean(Median)	8.8(9.0)	8.7(9.0)	n/a	0.8253	0.5506	12.5(12.0)	12.4(12.0)	n/a	2.4314	0.0252
Attention^a^	Mean(Median)	56.0(55.0)	56(55.0)	n/a	1.6195	0.1398	54.0(51.0)	53.9(51.0)	n/a	1.0493	0.3925
Internalizing^a^	Mean(Median)	60.2(61.0)	60(61.0)	n/a	0.3165	0.9284	60.0(61.0)	59.8(61.0)	n/a	0.7167	0.6363
Externalizing^a^	Mean(Median)	54.2(54.0)	54(54.0)	n/a	0.7558	0.6051	51.6(51.0)	51.3(51.0)	n/a	0.9881	0.4326
Total^a^	Mean(Median)	56.7(57.0)	56(57.0)	n/a	0.6031	0.7279	55.7(54.0)	55.4(54.0)	n/a	0.5637	0.7593

^a^CBCL for Class 2 to 4 and YSR for Class 5 to 7; ^**††**^Multivariate Pillai test

Table 2 summarizes comparison of mean pretest and posttest academic performance scores aggregate marks and per subject disaggregated by region for both primary and upper primary classes. There was significant improvement in all aggregate scores and in nearly all individual subjects, highlighted in bold. The only non-significant (p > 0.05) findings were on Science (rural lower classes and peri-urban upper classes), social studies (peri-urban upper classes) and Mathematics and Kiswahili - the national lingua franca (rural upper classes). There was significant change in all CBCL syndrome scores. The only non-significant change in YSR syndrome scores in were Attention and Externalizing (peri-urban).

**Table 2:** Academic performance and CBCL/YSR syndrome scores before and after intervention overall and by region

Subject & Syndrome	Time	Overall			Peri-Urban			Rural		
		N	Mean ± SD	P-Value^†^	N	Mean ± SD	P-Value^†^	N	Mean ±SD	P-Value^†^
Academic Performance and CBCL syndrome scores Before and After intervention Lower Classes (2-4)										
Aggregate	Before	976	244.1±70.8	<0.0001	547	243.3±68.9	<0.0001	429	245.2±73.2	<0.0001
marks	After		256.8±71.5			257.1±72.5			256.5±70.5	
English	Before	972	47.8±14.5	<0.0001	545	48.8±15.0	<0.0001	427	46.5±13.7	<0.0001
	After		51.1±16.2			52.5±16.5			49.4±15.6	
Kiswahili	Before	973	52.2±17.1	<0.0001	545	52.1±18.1	<0.0001	428	52.4±15.9	<0.0001
	After		56.2±16.7			56.4±17.2			55.9±16.1	
Mathematics	Before	973	47.5±19.1	0.0003	546	45.3±16.4	0.0090	427	50.3±21.7	0.0127
	After		49.3±18.5			47.0±18.0			52.1±18.7	
Science	Before	972	49.4±17.9	0.0126	546	49.3±16.6	0.0002	426	49.6±19.4	0.7402
	After		50.4±17.6			51.2±17.2			49.4±17.9	
Social	Before	967	47.0±15.8	<0.0001	541	47.7±15.9	<0.0001	426	46.1±15.5	<0.0001
Studies	After		50.5±15.8			50.4±16.3			50.7±15.3	
Attention^a^	Before	795	56.0±6.0	<0.0001	459	56.3±6.2	0.0232	336	55.6±5.8	<0.0001
	After		57.3±7.0			57.2±7.3			57.4±6.7	
Internalizing^a^	Before	794	60.3±10.8	<0.0001	458	60.3±10.7	0.0161	336	60.1±10.9	<0.0001
	After		62.2±7.2			61.7±7.4			63.0±6.7	
Externalizing^a^	Before	795	54.2±10.8	<0.0001	459	54.7±10.9	<0.0001	336	53.5±10.5	<0.0001
	After		57.9±7.2			57.4±7.1			58.4±7.3	
Total^a^	Before	795	56.7±11.6	<0.0001	459	57.2±11.8	<0.0001	336	56.1±11.4	<0.0001
	After		60.2±7.6			59.8±7.7			60.8±7.5	
Academic Performance and YSR syndrome scores Before and After intervention Upper Classes (5-7)										
Aggregate	Before	697	232.1±46.2	<0.0001	351	233.7±50.5	<0.0001	346	230.5±41.4	<0.0001
marks	After		243.4±48.7			240.6±52.9			246.2±43.8	
English	Before	695	47.2±10.6	<0.0001	350	48.3±11.7	0.0052	345	46.0±9.1	<0.0001
	After		49.9±10.2			49.5±11.4			50.2±8.8	
Kiswahili	Before	694	49.7±9.8	0.0006	351	49.4±10.9	0.0019	343	49.9±8.5	0.0812
	After		51.0±10.9			51.0±11.8			50.9±9.9	
Mathematics	Before	695	40.6±13.6	0.0001	350	36.9±12.5	0.0001	345	44.4±13.7	0.0958
	After		42.4±14.6			39.4±14.0			45.5±14.6	
Science	Before	696	48.5±14.6	<0.0001	350	49.8±15.7	0.1340	346	47.1±13.3	<0.0001
	After		50.7±14.6			50.8±15.6			50.6±13.4	
Social	Before	695	46.5±11.6	<0.0001	349	49.3±12.6	0.4229	346	43.6±9.9	<0.0001
Studies	After		49.2±13.1			49.7±13.4			48.6±12.8	
Attention^a^	Before	577	53.6±6.3	0.0013	295	54.1±6.6	0.0904	282	53.1±5.9	0.0030
	After		52.7±4.9			53.4±5.1			51.9±4.6	
Internalizing^a^	Before	577	59.5±10.2	<0.0001	295	60.6±9.6	<0.0001	282	58.4±10.7	<0.0001
	After		53.1±5.2			54.2±5.5			52.0±4.5	
Externalizing^a^	Before	577	50.8±11.5	0.0358	295	51.9±11.1	0.6989	282	49.5±11.8	0.0103
	After		51.8±4.3			52.2±4.6			51.3±4.0	
Total^a^	Before	577	55.0±11.7	<0.0001	295	56.5±11.1	<0.0001	282	53.3±12.1	0.0145
	After		52.4±4.8			53.1±5.2			51.6±4.3	

^†^CBCL for Class 2 to 4 and YSR for Class 5 to 7; ^a^Multivariate Pillai test

Table 3 summarizes the independent predictors of academic performance after the life-skills training intervention. All significant independent predictors of aggregate and individual subject scores in lower classes and upper classes are highlighted in bold.

**Table 3:** Independent Predictors of Academic performance in (Lower classes 2-4) and (Upper classes 5-7) after Intervention^a^

Parameter	Category	Aggregate	English	Kiswahili	Mathematics	Science	Social Studies
		β(95%C.I β)	β(95%C.I β)	β(95%C.I β)	β(95%C.I β)	β(95%C.I β)	β(95%C.I β)
Lower classes (2-4)							
Gender	Female	Ref.	Ref.	Ref.	Ref.	Ref.	Ref.
	Male	1.93(-3.55 to 7.41)	0.08(-1.69 to 1.86)	0.80(-1.33 to 2.92)	0.37(-2.00 to 2.74)	0.11(-1.96 to 2.18)	1.09(-0.92 to 3.11)
Guardianship	Both Parents	Ref.	Ref.	Ref.	Ref.	Ref.	Ref.
	Single parent	3.27(-7.34 to 13.88)	1.54(-1.91 to 4.98)	2.71(-1.40 to 6.83)	0.86(-3.73 to 5.46)	0.09(-3.93 to 4.10)	-2.63(-6.53 to 1.28)
	Others	-5.49(-19.89 to 8.91)	0.28(-4.39 to 4.96)	-3.9(-9.48 to 1.68)	-0.97(-7.21 to 5.26)	-1.64(-7.09 to 3.8)	0.64(-4.66 to 5.93)
Marital Status	Married	Ref.	Ref.	Ref.	Ref.	Ref.	Ref.
	Single/divorced	-4.01(-16.33 to 8.31)	-1.23(-5.23 to 2.77)	0.62(-4.16 to 5.39)	-1.90(-7.24 to 3.43)	-3.44(-8.10 to 1.21)	4.91(0.37 to 9.44)*
Fathers education level	Primary or less	Ref.	Ref.	Ref.	Ref.	Ref.	Ref.
	Secondary or more	11.86(2.93 to 20.79)**	2.92(0.02 to 5.82)*	1.58(-1.88 to 5.04)	3.43(-0.44 to 7.30)	-0.31(-3.69 to 3.07)	4.82(1.54 to 8.11)**
	Don’t know	10.11(0.41 to 19.81)*	1.73(-1.42 to 4.88)	2.50(-1.26 to 6.26)	-0.30(-4.50 to 3.90)	-0.47(-4.14 to 3.20)	4.51(0.94 to 8.08)*
Mothers education level	Primary or less	Ref.	Ref.	Ref.	Ref.	Ref.	Ref.
	Secondary or more	-5.31(-13.80 to 3.18)	-3.01(-5.77 to -0.26)*	-2.08(-5.37 to 1.21)	-0.03(-3.70 to 3.65)	1.72(-1.49 to 4.93)	-3.77(-6.89 to -0.64)*
	Don’t know	4.67(-4.42 to 13.76)	-2.20(-5.15 to 0.75)	-1.13(-4.65 to 2.40)	4.80(0.86 to 8.74)*	2.36(-1.08 to 5.80)	1.02(-2.32 to 4.36)
Fathers employment	Unemployed	Ref.	Ref.	Ref.	Ref.	Ref.	Ref.
	Employed	-2.08(-8.32 to 4.17)	-0.08(-2.11 to 1.95)	2.08(-0.34 to 4.50)	-2.31(-5.01 to 0.39)	-2.5(-4.86 to -0.14)*	0.30(-2.00 to 2.60)
Mothers employment	Unemployed	Ref.	Ref.	Ref.	Ref.	Ref.	Ref.
	Employed	2.12(-4.26 to 8.50)	-0.52(-2.59 to 1.55)	0.11(-2.37 to 2.58)	1.91(-0.85 to 4.67)	0.41(-2.01 to 2.82)	0.15(-2.20 to 2.50)
Region	Peri-Urban	Ref.	Ref.	Ref.	Ref.	Ref.	Ref.
	Rural	10.62(4.74 to 16.51)***	-0.37(-2.28 to 1.54)	0.18(-2.1 to 2.46)	7.29(4.74 to 9.84)***	0.41(-1.81 to 2.64)	2.93(0.77 to 5.10)**
Class	Two	Ref.	Ref.	Ref.	Ref.	Ref.	Ref.
	Three	-5.91(-13.44 to 1.62)	-0.78(-3.23 to 1.66)	-3.08(-6 to -0.17)*	-4.16(-7.42 to -0.90)*	0.8(-2.04 to 3.65)	-0.22(-2.99 to 2.55)
	Four	5.09(-3.61 to 13.80)	4.29(1.46 to 7.11)**	-5.85(-9.22 to -2.48)***	-1.51(-5.28 to 2.26)	3.93(0.64 to 7.22)*	3.62(0.42 to 6.82)*
Attention	Attention	-0.47(-0.99 to 0.04)	0.005(-0.16 to 0.17)	-0.01(-0.21 to 0.19)	-0.04(-0.27 to 0.18)	-0.28(-0.48 to -0.09)**	-0.13(-0.32 to 0.06)
Internalizing	Internalizing	-0.11(-0.64 to 0.41)	-0.06(-0.23 to 0.11)	0.06(-0.14 to 0.27)	0.003(-0.22 to 0.23)	-0.02(-0.22 to 0.18)	-0.04(-0.24 to 0.15)
Externalizing	Externalizing	-0.26(-0.79 to 0.26)	0.03(-0.15 to 0.20)	0.09(-0.11 to 0.30)	-0.17(-0.4 to 0.06)	-0.07(-0.27 to 0.13)	-0.09(-0.29 to 0.10)
Total Problems	Total	0.34(-0.58 to 1.25)	0.05(-0.25 to 0.35)	-0.20(-0.55 to 0.16)	0.11(-0.28 to 0.51)	0.16(-0.19 to 0.50)	0.12(-0.22 to 0.45)
Age in years	Age	-1.20(-3.45 to 1.05)	-0.63(-1.36 to 0.10)	-0.28(-1.15 to 0.59)	-0.20(-1.17 to 0.78)	-0.43(-1.28 to 0.42)	0.29(-0.54 to 1.12)
Upper classes (5-7)							
Gender	Female	Ref.	Ref.	Ref.	Ref.	Ref.	Ref.
	Male	1.95 (-3.04 to 6.93)	1.13 (-0.40 to 2.66)	-0.56 (-2.45 to 1.33)	1.56 (-0.74 to 3.85)	0.54 (-1.75 to 2.84)	0.10 (-1.70 to 1.90)
Guardianship	Both Parents	Ref.	Ref.	Ref.	Ref.	Ref.	Ref.
	Single parent	-5.34 (-15.07 to 4.39)	-2.76 (-5.75 to 0.23)	-4.72 (-8.40 to -1.04)*	0.85 (-3.62 to 5.33)	0.72 (-3.75 to 5.20)	0.09 (-3.42 to 3.61)
	Others	-7.53 (-19.19 to 4.12)	-3.25 (-6.83 to 0.32)	-3.04 (-7.45 to 1.37)	2.85 (-2.51 to 8.21)	-2.29 (-7.66 to 3.07)	-2.31 (-6.52 to 1.90)
Marital Status	Married	Ref.	Ref.	Ref.	Ref.	Ref.	Ref.
	Single/divorced	4.31 (-5.99 to 14.62)	0.69 (-2.47 to 3.85)	2.35 (-1.55 to 6.25)	-3.18 (-7.92 to 1.55)	3.48 (-1.26 to 8.22)	0.75 (-2.97 to 4.47)
Fathers education level	Primary or less	Ref.	Ref.	Ref.	Ref.	Ref.	Ref.
	Secondary or more	5.06 (-0.90 to 11.02)	1.62 (-0.21 to 3.45)	2.02 (-0.24 to 4.28)	1.12 (-1.63 to 3.86)	-0.83 (-3.57 to 1.91)	1.27 (-0.88 to 3.42)
	Don’t know	3.41 (-4.43 to 11.26)	-0.52 (-2.93 to 1.89)	1.64 (-1.33 to 4.61)	1.26 (-2.35 to 4.87)	-1.17 (-4.78 to 2.44)	2.67 (-0.17 to 5.50)
Mothers education level	Primary or less	Ref.	Ref.	Ref.	Ref.	Ref.	Ref.
	Secondary or more	-3.23 (-8.99 to 2.53)	-2.01 (-3.78 to -0.24)*	0.74 (-1.44 to 2.92)	-0.93 (-3.58 to 1.71)	0.42 (-2.23 to 3.07)	-0.90 (-2.98 to 1.18)
	Don’t know	0.38 (-7.17 to 7.94)	-1.04 (-3.36 to 1.28)	1.99 (-0.87 to 4.85)	-0.57 (-4.04 to 2.90)	-1.67 (-5.15 to 1.80)	1.10 (-1.62 to 3.83)
Fathers employment	Unemployed	Ref.	Ref.	Ref.	Ref.	Ref.	Ref.
	Employed	-0.52 (-5.98 to 4.95)	0.02 (-1.66 to 1.70)	0.46 (-1.61 to 2.53)	-0.27 (-2.78 to 2.25)	0.11 (-2.41 to 2.63)	-1.03 (-3.01 to 0.94)
Mothers employment	Unemployed	Ref.	Ref.	Ref.	Ref.	Ref.	Ref.
	Employed	-3.04 (-8.04 to 1.95)	-0.35 (-1.88 to 1.19)	-1.31 (-3.20 to 0.58)	-0.48 (-2.77 to 1.82)	-1.64 (-3.94 to 0.66)	1.28 (-0.52 to 3.08)
Region	Peri-Urban	Ref.	Ref.	Ref.	Ref.	Ref.	Ref.
	Rural	8.37 (3.28 to 13.46)**	2.73 (1.16 to 4.29)***	-1.85 (-3.78 to 0.07)	-1.30 (-3.64 to 1.04)	2.95 (0.61 to 5.30)*	5.63 (3.79 to 7.47) ***
Class	Five	Ref.	Ref.	Ref.	Ref.	Ref.	Ref.
	Six	-7.70 (-13.25 to -2.15)**	-3.80 (-5.51 to -2.10)***	1.53 (-0.57 to 3.63)	0.30 (-2.25 to 2.86)	-4.97 (-7.52 to -2.41)***	-0.89 (-2.89 to 1.12)
	Seven	2.61 (-6.69 to 11.91)	-0.20 (-3.06 to 2.65)	4.22 (0.70 to 7.74)*	3.14 (-1.14 to 7.42)	-4.20 (-8.48 to 0.08)	-1.49 (-4.85 to 1.87)
Attention	Attention	-0.43 (-0.85 to -0.002)*	-0.05 (-0.18 to 0.08)	-0.01 (-0.17 to 0.15)	-0.03 (-0.23 to 0.16)	-0.16 (-0.35 to 0.04)	-0.13 (-0.28 to 0.03)
Internalizing	Internalizing	-0.27 (-0.82 to 0.28)	-0.12 (-0.29 to 0.05)	-0.07 (-0.28 to 0.14)	-0.15 (-0.40 to 0.11)	0.04 (-0.21 to 0.29)	0.07 (-0.13 to 0.26)
Externalizing	Externalizing	0.10 (-0.38 to 0.58)	0.06 (-0.08 to 0.21)	-0.01 (-0.19 to 0.18)	-0.16 (-0.39 to 0.06)	0.11 (-0.11 to 0.33)	0.03 (-0.14 to 0.21)
Total Problems	Total	0.62 (-0.18 to 1.43)	0.16 (-0.09 to 0.41)	0.10 (-0.20 to 0.41)	0.19 (-0.18 to 0.56)	0.10 (-0.27 to 0.47)	0.002 (-0.29 to 0.29)
Age in years	Age	-3.06 (-5.23 to -0.9)**	-0.74 (-1.41 to -0.08)*	0.37 (-0.45 to 1.19)	-0.59 (-1.58 to 0.41)	-1.14 (-2.13 to -0.14)*	-0.64 (-1.43 to 0.14)

^a^ Results from multivariate model: Ref.-Reference category;β- Beta co-efficient is then the average difference in Academic performance scores between the category for the reference group and the category for which is the comparison group. *P < 0.05; **P < 0.01; ***P < 0.001

Table 4 summarizes the trends and significant change in academic performance after the life-skills training intervention adjusting for all the socio-demographics and CBCL indicators in the lower primary classes and YSR indicators in the upper primary classes. In almost all the scores (aggregate and individual subjects) in lower classes and upper classes, after controlling for other factors, the effect of time was significant in which there was increase in academic performance after intervention, highlighted in bold.

**Table 4:** Linear Mixed Model Assessing Predictors of Academic performance in (Lower classes 2-4) and (Upper classes 5-7) after Intervention^a^

Parameter	Category	Aggregate	English	Kiswahili	Mathematics	Science	Social Studies
		β(95%C.I β)	β(95%C.I β)	β(95%C.I β)	β(95%C.I β)	β(95%C.I β)	β(95%C.I β)
Lower classes (2-4)							
Gender	Female	Ref.	Ref.	Ref.	Ref.	Ref.	Ref.
	Male	-3.61(-9.96 to 2.74)	-3.98(-5.46 to -2.49)***	-3.18(-4.76 to -1.6)***	1.16(-0.61 to 2.93)	1.55(-0.22 to 3.32)	1.0(-0.60 to 2.60)
Guardianship	Both Parents	Ref.	Ref.	Ref.	Ref.	Ref.	Ref.
	Single parent	-11.61(-23.71 to 0.49)	-1.2(-4.02 to 1.61)	-4.28(-7.3 to -1.27)**	1.37(-4.73 to 1.99)	-1.85(-5.23 to 1.53)	-1.43(-4.44 to 1.58)
	Others	1.67(-14.41 to 17.74)	0.14(-3.61 to 3.89)	-1.59(-5.63 to 2.44)	3.89(-0.58 to 8.36)	-1.85(-6.34 to 2.63)	0.67(-3.35 to 4.69)
Marital Status	Married	Ref.	Ref.	Ref.	Ref.	Ref.	Ref.
	Single/divorced	4.0(-10.0 to 17.99)	-1.38(-4.65 to 1.88)	1.39(-2.08 to 4.87)	-0.22(-4.09 to 3.65)	3.2(-0.70 to 7.10)	-0.15(-3.66 to 3.33)
Fathers education level	Primary or less	Ref.	Ref.	Ref.	Ref.	Ref.	Ref.
	Secondary or more	0.98(-9.17 to 11.13)	-0.41(-2.76 to 1.93)	0.27(-2.23 to 2.77)	-1.52(-4.32 to 1.29)	2.4(-0.41 to 5.21)	0.44(-2.07 to 2.96)
	Don’t know	-0.78(-11.85 to 10.30)	-1.18(-3.75 to 1.39)	-0.77(-3.55 to 2.01)	-1.3(-4.38 to 1.78)	1.7(-1.40 to 4.79)	0.36(-2.40 to 3.13)
Mothers education level	Primary or less	Ref.	Ref.	Ref.	Ref.	Ref.	Ref.
	Secondary or more	2.86(-6.98 to 12.71)	0.57(-1.70 to 2.84)	1.04(-1.38 to 3.47)	0.56(-2.15 to 3.28)	0.04(-2.71 to 2.79)	1.30(-1.14 to 3.75)
	Don’t know	2.46(-8.12 to 13.04)	1.06(-1.39 to 3.51)	-0.36(-3.00 to 2.29)	0.07(-2.89 to 3.03)	0.07(-2.89 to 3.03)	1.75(-0.89 to 4.40)
Fathers employment	Unemployed	Ref.	Ref.	Ref.	Ref.	Ref.	Ref.
	Employed	-3.74(-11.12 to 3.63)	-1.08(-2.81 to 0.64)	-0.31(-2.14 to 1.53)	-1.65(-3.67 to 0.38)	-0.46(-2.52 to 1.60)	-0.69(-2.51 to 1.13)
Mothers employment	Unemployed	Ref.	Ref.	Ref.	Ref.	Ref.	Ref.
	Employed	6.65(-0.71 to 14.02)	1.76(0.03 to 3.49)*	0.99(-0.85 to 2.82)	0.33(-1.73 to 2.39)	2.06(-0.01 to 4.13)*	1.42(-0.42 to 3.27)
Region	Peri-Urban	Ref.	Ref.	Ref.	Ref.	Ref.	Ref.
	Rural	2.11(-13.55 to 17.19)	-2.59(-6.5 to 1.21)	-0.69(-3.82 to 2.27)	1.30(-1.16 to 10.71)	1.59(-2.28 to 5.67)	0.77(-2.79 to 4.24)
Class	Two	Ref.	Ref.	Ref.	Ref.	Ref.	Ref.
	Three	-11.84(-35.18 to 9.01)	1.33(-2.78 to 5.4)	1.25(-2.86 to 5.00)	-5.74(-11.93 to -0.07)	-2.34(-7.95 to 2.76)	-5.00(-9.37 to -0.89)*
	Four	-0.26(-20.53 to 18.2)	3.80(0.17 to 7.29)*	4.82(0.40 to 8.86)*	-5.51(-11.94 to 0.57)	-1.39(-6.84 to 3.64)	-0.51(-4.47 to 3.06)
Attention	Attention	-1.55(-2.33 to -0.78)***	-0.31(-0.49 to -0.13)***	-0.41(-0.60 to -0.22)***	-0.29(-0.50 to -0.08)**	-0.22(-0.43 to -0.003)*	-0.27(-0.47 to -0.08)**
Internalizing	Internalizing	0.39(-0.38 to 1.16)	0.22(0.04 to 0.4)*	0.02(-0.17 to 0.21)	0.05(-0.16 to 0.27)	0.06(-0.16 to 0.27)	0.07(-0.12 to 0.26)
Externalizing	Externalizing	-0.05(-0.87 to 0.77)	0.04(-0.15 to 0.23)	0.004(-0.21 to 0.2)	-0.05(-0.28 to 0.17)	-0.04(-0.27 to 0.19)	-0.04(-0.24 to 0.16)
Total Problems	Total	0.30(-1.08 to 1.68)	-0.12(-0.44 to 0.20)	0.14(-0.20 to 0.48)	0.10(-0.28 to 0.48)	0.09(-0.29 to 0.47)	0.1(-0.24 to 0.44)
Age in years	Age	-8.13(-10.67 to -5.58)***	-1.95(-2.55 to -1.36)***	-1.79(-2.43 to -1.16)***	-1.24(-1.95 to -0.53)***	-1.88(-2.6 to -1.17)***	-1.50(-2.14 to -0.86)***
Time	Pre-Post	14.09(8.08 to 20.1)***	3.60(2.20 to 4.99)***	4.26(2.76 to 5.75)***	1.30(-0.35 to 2.96)	1.25(-0.43 to 2.92)	3.99(2.48 to 5.49)***
Upper classes (5-7)							
Gender	Female	Ref.	Ref.	Ref.	Ref.	Ref.	Ref.
	Male	7.80(2.77 to 12.83)**	-0.10(-1.25 to 1.06)	-0.57(-1.76 to 0.63)	1.17(-0.38 to 2.73)	4.84(3.20 to 6.48)***	2.75(1.46 to 4.04)***
Guardianship	Both Parents	Ref.	Ref.	Ref.	Ref.	Ref.	Ref.
	Single parent	-5.28(-15.03 to 4.46)	-0.30(-2.53 to 1.92)	0.002(-2.30 to 2.31)	-1.94(-4.93 to 1.06)	-3.11(-6.29 to 0.07)	0.09(-2.44 to 2.62)
	Others	10.59(-1.13 to 22.31)	1.82(-0.86 to 4.51)	2.55(-0.23 to 5.34)	1.97(-1.65 to 5.58)	1.65(-2.18 to 5.48)	3.10(0.10 to 6.10)*
Marital Status	Married	Ref.	Ref.	Ref.	Ref.	Ref.	Ref.
	Single/divorced	14.99(4.77 to 25.21)**	2.88(0.54 to 5.21)*	2.34(-0.07 to 4.76)	2.01(-1.14 to 5.17)	5.19(1.85 to 8.54)**	2.21(-0.42 to 4.84)
Fathers education level	Primary or less	Ref.	Ref.	Ref.	Ref.	Ref.	Ref.
	Secondary or more	3.49(-2.49 to 9.46)	0.92(-0.45 to 2.29)	0.23(-1.20 to 1.66)	0.81(-1.03 to 2.66)	0.79(-1.17 to 2.75)	0.43(-1.11 to 1.97)
	Don’t know	-7.34(-15.34 to 0.65)	-0.87(-2.71 to 0.97)	-0.82(-2.74 to 1.10)	-2.07(-4.54 to 0.40)	-1.86(-4.48 to 0.75)	-2.10(-4.16 to -0.05)*
Mothers education level	Primary or less	Ref.	Ref.	Ref.	Ref.	Ref.	Ref.
	Secondary or more	3.46(-2.39 to 9.31)	0.76(-0.57 to 2.09)	0.70(-0.68 to 2.08)	0.96(-0.82 to 2.74)	-0.21(-2.11 to 1.69)	0.98(-0.53 to 2.48)
	Don’t know	1.88(-6.11 to 9.87)	-1.03(-2.86 to 0.79)	-0.28(-2.18 to 1.62)	2.67(0.20 to 5.13)*	-0.01(-2.64 to 2.62)	0.45(-1.61 to 2.52)
Fathers employment	Unemployed	Ref.	Ref.	Ref.	Ref.	Ref.	Ref.
	Employed	-3.60(-9.21 to 2.02)	-0.74(-2.02 to 0.54)	-0.35(-1.68 to 0.99)	-2.04(-3.76 to -0.31)*	-1.27(-3.11 to 0.57)	0.53(-0.91 to 1.98)
Mothers employment	Unemployed	Ref.	Ref.	Ref.	Ref.	Ref.	Ref.
	Employed	1.24(-3.86 to 6.35)	-0.06(-1.22 to 1.11)	0.29(-0.93 to 1.50)	1.26(-0.32 to 2.84)	0.26(-1.41 to 1.94)	0.11(-1.20 to 1.42)
Region	Peri-Urban	Ref.	Ref.	Ref.	Ref.	Ref.	Ref.
	Rural	28.33(6.82 to 50.09)***	1.8(-1.99 to 5.66)	1.94(-1.68 to 5.74)	11.61(6.43 to 16.60)***	3.6(-2.82 to 9.94)	0.08(-6.11 to 6.45)
Class	Five	Ref.	Ref.	Ref.	Ref.	Ref.	Ref.
	Six	11.15(1.65 to 21.26)*	2.74(0.51 to 5.00)*	-0.13(-2.52 to 2.41)	4.78(1.99 to 7.59)**	1.70(-2.38 to 5.81)	2.32(-0.05 to 4.94)
	Seven	24.42(7.52 to 40.86)**	4.56(0.76 to 8.29)*	1.99(-2.00 to 6.15)	9.28(4.94 to 13.51)***	5.11(-0.96 to 10.95)	4.27(0.13 to 8.54)*
Attentions problems	Attention	-0.82(-1.39 to -0.25)**	-0.15(-0.29 to -0.02)*	-0.18(-0.32 to -0.05)**	-0.19(-0.37 to -0.02)*	-0.18(-0.37 to 0.002)	-0.09(-0.24 to 0.06)
Internalizing problems	Internalizing	0.35(-0.42 to 1.12)	0.09(-0.09 to 0.27)	0.08(-0.1 to 0.27)	0.22(-0.03 to 0.46)	0.004(-0.25 to 0.26)	-0.01(-0.21 to 0.19)
Externalizing problems	Externalizing	-0.72(-1.4 to -0.05)*	-0.17(-0.32 to -0.02)*	-0.03(-0.19 to 0.14)	0.03(-0.18 to 0.24)	-0.27(-0.49 to -0.05)*	-0.21(-0.38 to -0.04)*
Total Problems	Total	-0.07(-1.23 to 1.1)	-0.04(-0.3 to 0.23)	-0.11(-0.38 to 0.17)	-0.2(-0.56 to 0.16)	0.06(-0.32 to 0.44)	0.11(-0.19 to 0.41)
Age in years	Age	-8.71(-11.01 to -6.42)***	-1.83(-2.36 to -1.31)***	-1.37(-1.92 to -0.82)***	-2.00(-2.7 to -1.29)***	-1.77(-2.52 to -1.02)***	-1.61(-2.2 to -1.02)***
Time	Pre-Post	11.44(6.06 to 16.82)***	2.61(1.38 to 3.84)***	0.91(-0.37 to 2.19)	2.44(0.76 to 4.11)**	2.41(0.65 to 4.16)**	2.8(1.42 to 4.17)***

^a^ Results from a single mixed model adjusting for all response variables allowing intercept and class grade to vary by school group. : *Ref.*-Reference category;*β*- Beta coefficient is then the average difference in Academic performance scores between the category for the reference group and the category for which the comparison group; *P < 0.05; **P < 0.01; ***P < 0.001

As shown in Table 5 on correlations, there was positive association between improvement in aggregate scores; English and science scores with improvement in internalizing, externalizing and total problems. However, there was no association between academic improvement and attention problems. The same was also replicated when regressing academic performance and individual syndromes controlling for gender, guardianship, marital status, father education level, mother education level, father employment, mother employment, region, class and age. However, when all the syndromes were added to the regression model (Model 5), there was loss of this association except for attention problem on aggregate scores (*p *< 0.05).

**Table 5:** Correlation between Academic Improvement and Symptom Reduction on Specific Problems and specific symptom reduction associated with improvement in Academic performance

A) Correlation between Academic Improvement and Symptom Reduction on Specific Problems						
Pearson’s Correlation	English	Kiswahili	Mathematics	Science	Social Studies	Aggregate Marks
a) Attention Problems	0.056	0.032	-0.062	0.03	-0.029	0.004
b) Internalizing Problems	**0.121** ^**^	0.01	-0.04	**0.135** ^**^	0.037	**0.113** ^**^
c) Externalizing Problems	**0.159** ^**^	0.027	**-0.093** ^*^	**0.145** ^**^	0.02	**0.122** ^**^
d) Total Problems	**0.153** ^**^	0.025	-0.062	**0.133** ^**^	0.019	**0.122** ^**^
B) **Coefficients from Regression of Academic Improvement and Symptom Reduction on Specific Problems**						
** Aggregate Scores**	**Model 1**	**Model 2**	**Model 3**	**Model 4**	**Model 5**	
a) Attention Problems	-0.01(0.18)				-0.43*(0.22)	
b) Internalizing Problems		0.29*(0.13)			-0.27(0.28)	
c) Externalizing Problems			0.41***(0.11)		0.10(0.25)	
d) Total Problems				0.38***(0.12)	0.62(0.41)	
**Science scores**	**Model 1**	**Model 2**	**Model 3**	**Model 4**	**Model 5**	
a) Attention Problems	0.01(0.08)				-0.16(0.10)	
b) Internalizing Problems		0.16**(0.06)			0.04(0.13)	
c) Externalizing Problems			0.17***(0.05)		0.11(0.11)	
d) Total Problems				0.16**(0.05)	0.10(0.19)	
**English Scores**	**Model 1**	**Model 2**	**Model 3**	**Model 4**	**Model 5**	
a) Attention Problems	0.07(0.05)				-0.05(0.07)	
b) Internalizing Problems		0.08*(0.04)			-0.12(0.09)	
c) Externalizing Problems			0.12***(0.03)		0.06(0.08)	
d) Total Problems				0.11***(0.03)	0.16(0.13)	

Coefficients for Academic achievement gains scores are from generalized linear models; Standard errors are in the parentheses; All models included controls for gender, guardianship, marital status, father education level, mother education level, father employment, mother employment, region, class and age; *P < 0.05; **P < 0.01;***P < 0.001

## Discussion

This is the first study in Kenya showing improvements in aggregate educational scores, English and science scores on internalizing, externalizing and total problems. It is also the first to demonstrate the impact of life-skills training, on mental health and academic performance in a Kenyan setting. An incidental positive finding of this study is that in Kenya, girls and boys are receiving equal access to education in both rural and urban areas (Table 1). We attribute this equity to the compulsory education for all children in Kenya.

There are some common findings with our and HIC studies. Firstly, attention problems were associated with least improvements even on interventions as has been found elsewhere [1, 9, 30, 31]. This is not surprising given that attention problems, regardless of whether they occur in LMICs or HICs, are associated with cognitive dysfunctions and organic brain syndromes [32, 33] and therefore less amenable to life skills training than other syndromes. This study population was drawn from LMIC where it is expected there are higher levels of malnutrition and in particular an average of 42% in Makueni County of stunted growth related to malnutrition which can be expected to result in and brain insults[34]. Another agreement with findings from HICs is that mental health syndromes that are amenable to interventions were associated with best academic outcomes confirming that mental disorders are associated with academic performance [4, 35–37]. Our findings further agree with findings from HICs that early treatment of mental disorders in children may improve academic performance [11, 38]. The urgency for intervention in LMICs is the same as in HICs given similar epidemiological patterns and prevalence of various mental disorders as pointed out under the Introduction. School forums allow for reach to critical numbers at much younger age, make maximal use of the resources that are available and afford more children the opportunity to manipulate and improve their trajectories in later life. We have demonstrated the feasibility of this [23, 39]. The fact that we used 2 different cohorts – rural and urban and demonstrated similar trends on outcomes on different syndromes points to the success of the interventions. We speculate that parental education (father and mother) was associated with better academic performance at lower classes (and therefore lower ages) because at lower age the children most important environment are the parents while at higher classes the physical environment becomes more noticeable by the children.

These feasibility findings have a particular relevance to a LMIC where there is a dearth of mental health expertise. The fact that these skills can be administered by teachers already operational within the existing system has implications for sustainability of the intervention using already existing resources. This is a good example of the task shifting model where complicated intervention best administered by highly qualified experts can be administered by teachers who have been trained on the administration of the skills

### Strengths

There were high follow up rates (83.2 – 95.4%) which we attribute to minimal change of schools in the course of the study, also a reflection of the students’ interest in participation in the study and the support of the parents and the teachers for this study.

#### Limitation

The interpretations of our findings are limited in several ways. We have no prior data from Africa to compare with. Secondly, the outcomes between lower primary and upper primary classes are limited for purposes of comparison because the measures are different and by different participants in each year group. CBCL for lower primary relied on parent’s perception of problems and YSR for upper children was by the children themselves on how they perceived their own problems. Many studies from across the globe indicate disagreement between parents rating their children using CBCL and children rating themselves using YSR [40–45]. Other studies suggest some agreements on CBCL by parents and YSR [46, 47]. However, this is only applicable if there is the same cohort of parents and same cohort of students both focusing on the students at more or less the same time. Furthermore, the adapted versions for the life skills are different for lower primary and upper primary classes as they were designed to be age appropriate.

We employed a single group pretest and posttest design without a control group or waitlisted group for comparison. However, this limitation is mainly logistical to separate children in the same class to one group receiving an intervention and another one not receiving intervention – can have negative impacts – those receiving intervention being viewed as the ones with mental illness by those not receiving intervention. We used a subsample of Kenya, so our results are not necessarily representative of all Kenyan students. The socio-demographics of the guardians/ parents were given by the children. This most likely explains the high levels of missing information (7.8 – 12.8%) in both rural and peri-urban schools since the children may not be in the know of all socio-demographics of their parents.

## Conclusions

Our study is an additional demonstration of the feasibility of life-skills training, in this case with the focus on academic improvement, probably mediated by improvements in mental health. We advocate for further mixed methods studies that will take into account these preliminary findings, to determine more precisely the design and then deliver randomized clinical trials to test school based interventions for mental health for impact on childhood mental health and academic performance.

## Data Availability

The datasets used and/or analysed during the current study are available from the corresponding author on reasonable request.
